# Tree Size Influences Leaf Shape but Does Not Affect the Proportional Relationship Between Leaf Area and the Product of Length and Width

**DOI:** 10.3389/fpls.2022.850203

**Published:** 2022-06-09

**Authors:** Jianzhong Ma, Karl J. Niklas, Leyi Liu, Zhendong Fang, Yirong Li, Peijian Shi

**Affiliations:** ^1^Yunnan Academy of Forestry and Grassland, Kunming, China; ^2^Bamboo Research Institute, Nanjing Forestry University, Nanjing, China; ^3^Plant Biology Section, School of Integrative Plant Science, Cornell University, Ithaca, NY, United States; ^4^College of Landscape Architecture and Horticulture Science, Southwest Forestry University, Kunming, China; ^5^Shangri-la Alpine Botanical Garden, Shangri-la, China

**Keywords:** bilateral symmetry, centroid ratio, DBH, growth patterns, leaf ellipticalness index, Montgomery equation

## Abstract

The Montgomery equation predicts leaf area as the product of leaf length and width multiplied by a correction factor. It has been demonstrated to apply to a variety of leaf shapes. However, it is unknown whether tree size (measured as the diameter at breast height) affects leaf shape and size, or whether such variations in leaf shape can invalidate the Montgomery equation in calculating leaf area. Here, we examined 60 individual trees of the alpine oak (*Quercus pannosa*) in two growth patterns (trees growing from seeds vs. growing from roots), with 30 individuals for each site. Between 100 and 110 leaves from each tree were used to measure leaf dry mass, leaf area, length, and width, and to calculate the ellipticalness index, ratio of area between the two sides of the lamina, and the lamina centroid ratio. We tested whether tree size affects leaf shape, size, and leaf dry mass per unit area, and tested whether the Montgomery equation is valid for calculating leaf area of the leaves from different tree sizes. The diameters at breast height of the trees ranged from 8.6 to 96.4 cm (tree height ranged from 3 to 32 m). The diameter at breast height significantly affected leaf shape, size, and leaf dry mass per unit area. Larger trees had larger and broader leaves with lower leaf dry mass per unit area, and the lamina centroid was closer to the leaf apex than the leaf base. However, the variation in leaf size and shape did not negate the validity of the Montgomery equation. Thus, regardless of tree size, the proportional relationship between leaf area and the product of leaf length and width can be used to calculate the area of the leaves.

## Introduction

Leaf shape has been demonstrated to be important for light interception, evapotranspiration, and mechanics (Niklas, 1988, 1999; Nicotra et al., 2008, 2011), and thus to affect the tradeoff between the leaf support cost and photosynthetic returns (Niinemets et al., 2007; Lin et al., 2020). For example, using computer simulations, Niklas (1988, 1989) reported that the extent of leaf lobing affected the capacity to intercept light, whereas Santiago and Kim (2009) found that *Sonchus* species from exposed habitats have smaller, more dissected leaves with greater photosynthetic rates compared with those of *Sonchus* species from shaded habitats. Thus, leaf shape can often be used as a predictor of photosynthetic capacity of leaves, such as rates of carbon uptake (Ölçer et al., 2001; Royer and Wilf, 2006). In this context, Shi et al. (2021a) showed that the ratio of leaf width to length (RWL) is significantly positively correlated with the fractal dimension of leaf shape, which means that RWL is a good indicator of the geometric characteristics of leaf shape. Using 101 bamboo taxa, Lin et al. (2020) demonstrated that the scaling exponent of leaf dry mass vs. leaf surface area decreases toward 1 as RWL increases, thereby indicating that broader leaves tend to have lower support cost with increasing unit leaf area compared to narrower leaves.

In addition to RWL, other leaf shape indices are available, i.e., the leaf roundness index and its reciprocal, the leaf dissection index (Kincaid and Schneider, 1983; Thomas and Bazzaz, 1996; Niinemets, 1998; Santiago and Kim, 2009; Peppe et al., 2011). However, an accurate quantification of many elliptical, oval, and oboval leaves significantly deviates from circular leaves. Consequently, Li et al. (2021b) proposed a new index, the leaf ellipticalness index (EI), based on the Montgomery equation (ME; see Montgomery, 1911), which assumes that leaf area is proportional to the product of leaf length and width. In contrast to the leaf roundness index, the EI reflects the extent to which an elliptical leaf deviates from an ellipse, and can be used to calculate leaf area provided that leaf length and width are known. In theory, the EI value can be larger or smaller than 1 depending on leaf shape. It cannot be used to accurately evaluate the degree of leaf bilateral asymmetry, or predict the leaf centroid from the base of an oval or oboval leaf shape. In order to cope with this limitation, Shi et al. (2021b) developed an ovate and obovate leaf shape model using leaf length and width and a third parameter representing the distance from the leaf base to the point on the leaf length axis associated with maximum leaf width. Consequently, Li et al. (2021c) defined the “centroid ratio” (as the ratio of this third parameter to leaf length) to quantify the extent of the deviation of the leaf centroid from the midpoint of leaf length. Using this model, Li et al. (2021c) found that the centroid ratio is significantly correlated with the ratio of leaf petiole mass to lamina mass for two Lauraceae species (*Cinnamomum camphora*, and *Machilus leptophylla*). Therefore, the centroid ratio is a potentially a good quantitative index of leaf shape. It is necessary to point out the difference between the centroid ratio (as the ratio of the distance, from the leaf base to a point on the leaf length axis associated with the maximum leaf width, to the leaf length) and centroid size in geometric morphometrics (Mitteroecker et al., 2013; Klingenberg, 2016). The latter is equal to the Euclidean distance between the landmarks on the boundary of a planar polygon to their centroid, which the centroid is the geometric centre of the polygon. In the present work, the definition of the “centroid” is the point on the leaf length axis associated with the maximum leaf width, which is not the geometric center in geometric morphometrics methods. The reason is that it is difficult to find landmarks on the boundary of a completely or approximately entire leaf.

A critical and as yet unanswered question is whether plant size (which is often but not invariably correlated with the age of perennial plant species) affects leaf shape or size. Tree populations usually consist of different age- and size-groups. For evergreen tree species, leaves are in a constant state of renewal, and limited research has shown that leaf and overall plant age can to a large extent determine overall photosynthetic capacity (Küppers, 1989; Bielczynski et al., 2017). In addition, tree height, which is often correlated with age, is important because water transport from roots to the highest elevated leaves becomes progressively more difficult (Becker et al., 2000). Thus, the leaf size, shape, leaf-level cost of light-interception (which can be quantified by leaf dry mass per unit area, LMA, or its reciprocal specific leaf area, SLA) can vary significantly within a canopy (Sack et al., 2006; He and Yan, 2018). Nevertheless, whether leaf shape varies significantly across tree size has not been tested.

**Figure 1:**
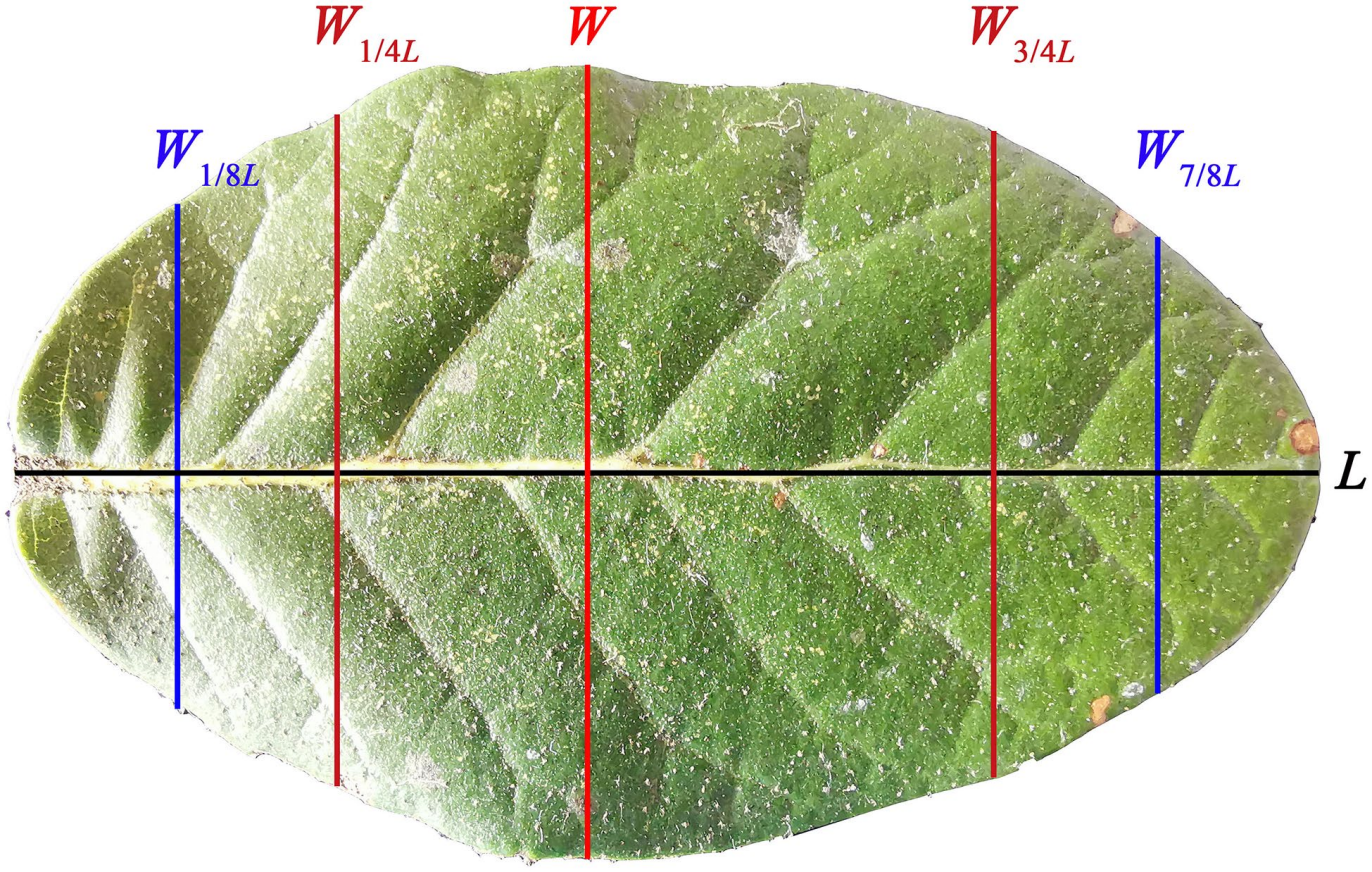
Adaxial surface (i.e., the upper image) of a representative leaf of *Quercus pannosa*. *L* represents the leaf length; *W* represents the maximum leaf width; *W*_1/4*L*_ represents the leaf width associated with 1/4 *L* from leaf base; *W*_3/4*L*_ represents the leaf width associated with 3/4 *L* from leaf base; *W*_1/8*L*_ represents the leaf width associated with 1/8 *L* from leaf base; *W*_7/8*L*_ represents the leaf width associated with 7/8 *L* from leaf base.

*Quercus pannosa* was selected for study because it is an important evergreen tree species, which usually forms a single forest or a mixed forest with other *Quercus* species typically growing at altitudes of 3,300–4,200 m in China. The species also produces leaves that are elliptical or oboval in shape (Figure 1) with a high dry mass per unit area (LMA). The leaf structure and shape of this species allows it to tolerate low temperatures and to grow closer to the climatic conifer treeline (He et al., 1994; Yang et al., 2020). He et al. (1994) explored the relationship between leaf anatomical structures and elevation of alpine oaks, and found that at high altitudinal areas the quadrangular and pentagonal epidermis in leaves are frequently observed, and that the stomatal density decreases at high elevations. However, there are no studies that have quantified the leaf-shape of this species, or that have related leaf-shape and LMA to tree size.

To address this question, we sampled >6,500 leaves from 60 naturally growing individual trees of *Quercus pannosa* Hand.-Mazz. at two sites (representing two growth patterns, i.e., trees growing from seeds vs. trees growing from roots) in south-western China to test: (i) whether tree size and growth pattern affects leaf shape, size, and leaf-level cost of light interception, (ii) whether the ME is valid for calculating the leaf area of different tree sizes at the individual tree level and for the pooled data across all individuals, and (iii) whether the EI differs from other leaf-shape indices including the leaf RWL and centroid ratio. In general, although the diameter at breast height (DBH) is positively correlated tree height, in practice, DBH is easier to accurately measure tree size. Therefore, in the present study, DBH is used as a measure of tree size.

## Materials and Methods

### Sampling Sites and Leaf Collection

Two sites (see Table 1 for details) measuring 100 m × 100 m in naturally growing tree communities were selected for study in Shangri-la, Yunnan Province, China. The annual accumulated precipitation for Shangri-la from 2000 to 2019 is 624 ± 124 mm; the mean annual temperature is 6.8°C ± 0.4°C; the annual duration of sunshine is 2,182 ± 149 h; the number of days for frosts per year is 152 ± 12 days (China Meteorological Data Service Centre).^1^ Thirty trees were randomly selected from the first site (S1), and another 30 trees were randomly selected from the second site (S2). For S1, *Q. pannosa* was intermixed with *Q. pseudosemecarpifolia*, and the coverage of either oak species accounted for *ca.* 25%–35%. For S2, *Q. pannosa* dominated the forest community, and accounted for *ca.* 85%–95% of the total forest coverage. Most trees grew from seeds in S1; most trees in S2 grew from roots. Most trees growing from seeds in S2 were cut down by local farmers, and the following trees growing from roots were usually shorter and the trunks near the ground are most curved, which is easy to distinguish between the trees of two growing patterns by observing tree size and simultaneously checking how bent the trunks are. In addition, S1 is far away from villages, and it is difficult for local farmers to arrive; however, S1 is closer to villages, and local farmers used to go to this site and the surrounding area to cut firewood. There were 22 out of the 30 trees whose DBH values ≥ 30 cm in S1, but were only 2 out of the 30 trees whose DBH values ≥ 30 cm in S2. Our experimental design is to choose 30 trees from each site, representing the smallest big sample size in statistics. In each site, we randomly sampled 30 trees in the range of 100 m × 100 m, and there is no need to sample more trees given the heavy workload required. We used a quadrat of 20 m × 20 m around the center of each site to measure the site information (Table 1).

**Table 1:** Site information.

Information	Site 1 (S1)	Site 2 (S2)
Location	27° 37′33.05″	99°34′0.68″
Elevation (m)	3,202	3,716
Size (m^2^)	20 × 20	20 × 20
Slope (°)	27	24
Aspect (°)	172	158
Soil type	Brown soil	Brown soil
Soil depth (m)	0.5–1.0	0.1–0.2
Dominant species of the community	*Quercus pannosa* *Quercus pseudosemecarpifolia* *Rhododendron decorum* *Fragaria nilgerrensis*	*Quercus pannosa* *Rhododendron Rubiginosum* *Ainsliaea fragrans*
Coverage of *Q. pannosa* (%)	25–35	85–95
Human disturbance	Weak	Strong
Main growth pattern of *Q. pannosa*	Growing from seeds	Growing from roots

*The trees are all native species for both S1 and S2, and are naturally distributed in Site 1. Local farmers often cut down trees in and around S2 for daily use. However, it is difficult to accurately estimate tree age for each individual. We randomly sampled 30 trees from each site of 100 m × 100 m, and we calculated the site information in this table using one quadrat of 20 m × 20 m around the center of each site*.

On 25 September 2021, we randomly sampled 100–110 leaves from the lower canopy of each of the 30 trees in S1, and on 1 October 2021, we sampled leaves from S2. Because of the difference in height among different individual trees, we defined “the lower canopy” as the positions of ≤1/4 of a tree crown height, and sampled leaves without distinguishing directions and between the shade and sun leaves given that a large sample can well reflect general characteristics of leaf shape and size. All leaves were wrapped in wet newspaper to reduce tissue dehydration.

### Indices for Measuring Leaf Shape

To quantify leaf shape, we used six indices.

(i) The ratio of leaf width to length (RWL)


RWL=W/L,

where *W* denotes leaf maximum width, and *L* denotes leaf length.

(ii) The leaf ellipticalness index (EI; Li et al., 2021a,b)


$$\mathrm{EI}=\frac{A}{\left(\pi /4\right)LW},$$

where *A* denotes leaf area.

(iii) The ratio of the *W* associated with 1/4 *L* from leaf base to the leaf width associated with 3/4 *L* from leaf base, which is referred to as the proximal ratio index (PRI). To normalize this parameter, we used its log-transformed value, i.e.,


$$\ln\mathrm{PRI}=\ln\frac{W_{1/4L}}{W_{3/4L}}.$$

(iv) The ratio of the *W* associated with 1/8 *L* from the leaf base to the width associated with 7/8 *L* from the leaf base. This ratio is referred to as the distal ratio index (DRI). To normalize this parameter, we also used its log-transformed value, i.e.,


$$\ln\mathrm{DRI}=\ln\frac{W_{1/8L}}{W_{7/8L}}$$

(v) The area ratio of the two sides of a leaf (AR). We used a log-transformed form to normalize this parameter, i.e.,


$$\ln\mathrm{AR}=\ln\frac{A_{\mathrm{Left}}}{A_{\mathrm{Right}}},$$

where $A_{\mathrm{Left}}$ and $A_{\mathrm{Right}}$ represent the areas of the left and right sides of a leaf, respectively.

(vi) The centroid ratio (CR), which is the ratio of the distance from leaf base to a point on the leaf length axis associated with leaf maximum width (*L_W_*) to leaf length (*L*), i.e.,


$$\mathrm{CR}=\frac{L_{W}}{L}.$$

We did not take mathematically transformed forms (i.e., the log-transformation) of RWL, EI, or CR because the log-transformation did not improve the normality of these data, and because the tails of the histograms of those variables did not exhibit skewness.

### Image Processing and Data Acquisition

After taking leaves back to the laboratory of Shangri-la Alpine Botanical Garden, we used three photo scanners (Type: CanoScan LiDE 220, Cannon, Vietnam) to scan all leaves to JPE images at 600 dpi resolution. The leaves were then dried using an oven (DHG 9070A, SoodKing, Suzhou, China) at 108°C for 48 h until achieving constant dry mass. We used an electric balance (BSA 124S, Sartorius Scientific Instruments Ltd., Beijing, China; measurement accuracy: 10^−4^ g) to measure leaf dry mass.

The scanned images were transformed to black–white BMP images, and we used the protocols proposed by Shi et al. (2018) to obtain the planar coordinates of each leaf edge. We used the statistical software R (version 4.2.0; R Core Team, 2022) to run the R script developed by Su et al. (2019) to calculate leaf area, length and width. To calculate PRI, DRI and CR, the slightly modified R script of Su et al. (2019) was used to provide values, which has been combined into the “bilat” function in a special R package “biogeom” (Shi et al., 2022a) was used to calculate the parameters related to leaf shape and size.

### Statistical Analyses

The ANOVA followed by the Tukey’s honest significant difference (HSD) test at the 0.05 significance level (Hsu, 1996) was used to test the significance of the differences between any two individual trees in their leaf size, shape, and LMA.

To check the influence of DBH on leaf shape, size, and LMA, linear mixed-effects models (Bates et al., 2015) were used. For each tree, there was one DBH value, and 100–110 measurements for leaf shape, size, and LMA (i.e., those of 100–100 leaves). DBH was regarded as a fixed effect, and site (representing the levels of the two growth patterns, i.e., trees from seeds vs. trees from roots) as a random effect. The intraclass correlation coefficient was calculated to check the extent of variation between the levels:


$$\rho =\frac{\sigma_{\alpha }^{2}}{\sigma_{\alpha }^{2}+\sigma_{\varepsilon }^{2}},$$

where *σ_α_* and *σ_ε_* represent the standard errors between the levels and within the levels, respectively. When there is no variation between the levels, *σ_α_* = 0 and *ρ* = 0; when the variation between the levels is much larger than that within the levels, *ρ* will approach 1 (Faraway, 2006).

To check whether tree size affected leaf area, the Montgomery equation (ME; Montgomery, 1911) was used:


A=MP⋅LW,

where MP is the Montgomery parameter, i.e., the proportionality coefficient to be estimated. We used the log-transformation of this equation to stabilize the variance of leaf area, i.e.,


lnA=a+lnLW,

where *a* is the natural logarithm of MP. When the ME held true, EI could be used as an indicator of leaf shape (Li et al., 2021b). The MP has a relationship with EI as:


$$\mathrm{MP}=\frac{\pi }{4}\mathrm{EI}.$$

According to the principle of similarity (Thompson, 1917), the area of an object is usually proportional to the square of its length. However, the empirical estimates for the scaling exponent of leaf area vs. leaf length for complex leaf shapes (especially those with lobes) can deviate from 2 (Shi et al., 2019; Yu et al., 2019, 2020). However, for elliptical, oval, and oboval leaf shapes, the principle of similarity has been confirmed (Shi et al., 2022b). Because the leaves of *Q. pannosa* exhibit elliptical and oboval shapes, it was nevertheless necessary to check whether it follows the principle of similarity. If and when it is confirmed, it can simplify the calculation of leaf area only using one leaf length dimension. We also checked whether the extent of variation in RWL influenced the validity of the principle of similarity. We calculated the root-mean-square error (RMSE) of fitting the following equation


lnA=c+2lnL,

and checked whether RMSE increases with the increase of the coefficient of variation (CV) in RWL. If it increases with increasing CV in RWL, it signifies the extent to which the principle of similarity depends on the variation in RWL. All analyses were carried out using the statistical software R (version 4.2.0; R Core Team, 2022).

## Results

**Figure 2:**
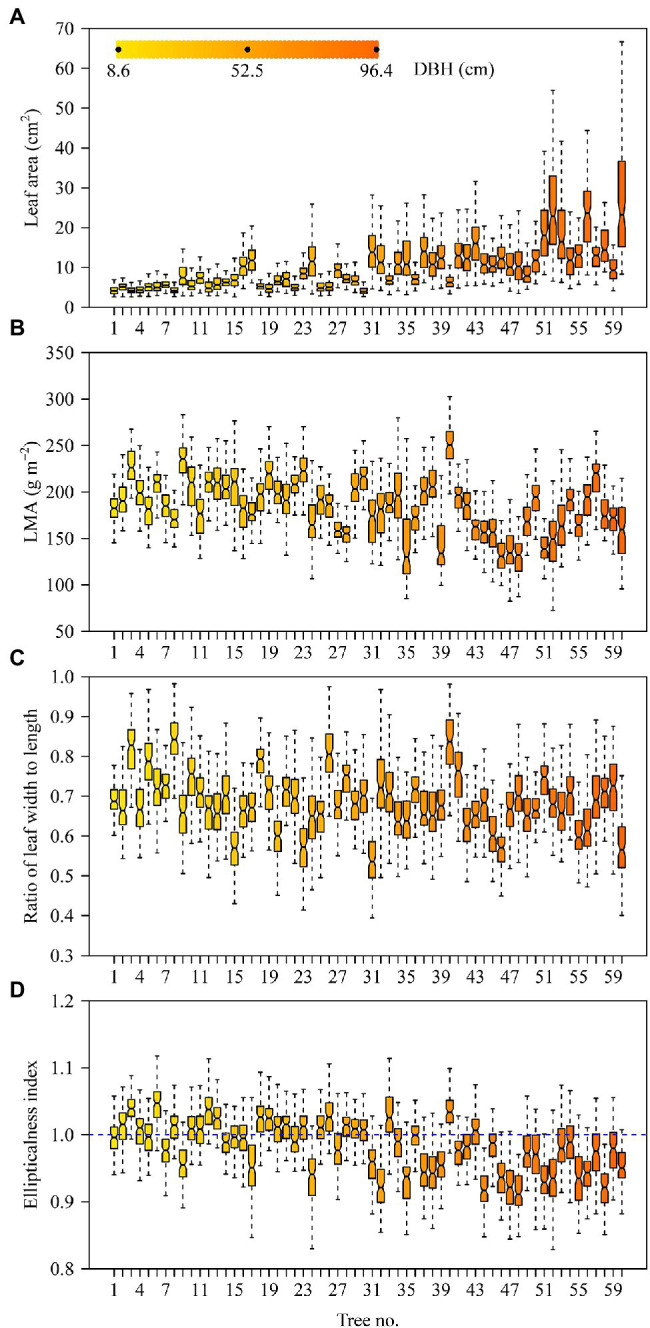
Boxplots of leaf area **(A)**, leaf dry mass per unit area **(B)**, ratio of leaf width to length **(C)**, and leaf ellipticalness index **(D)**. The notches of boxes represent the medians, and the colors of boxes reflect their DBH values (see panel **A**).

**Figure 3:**
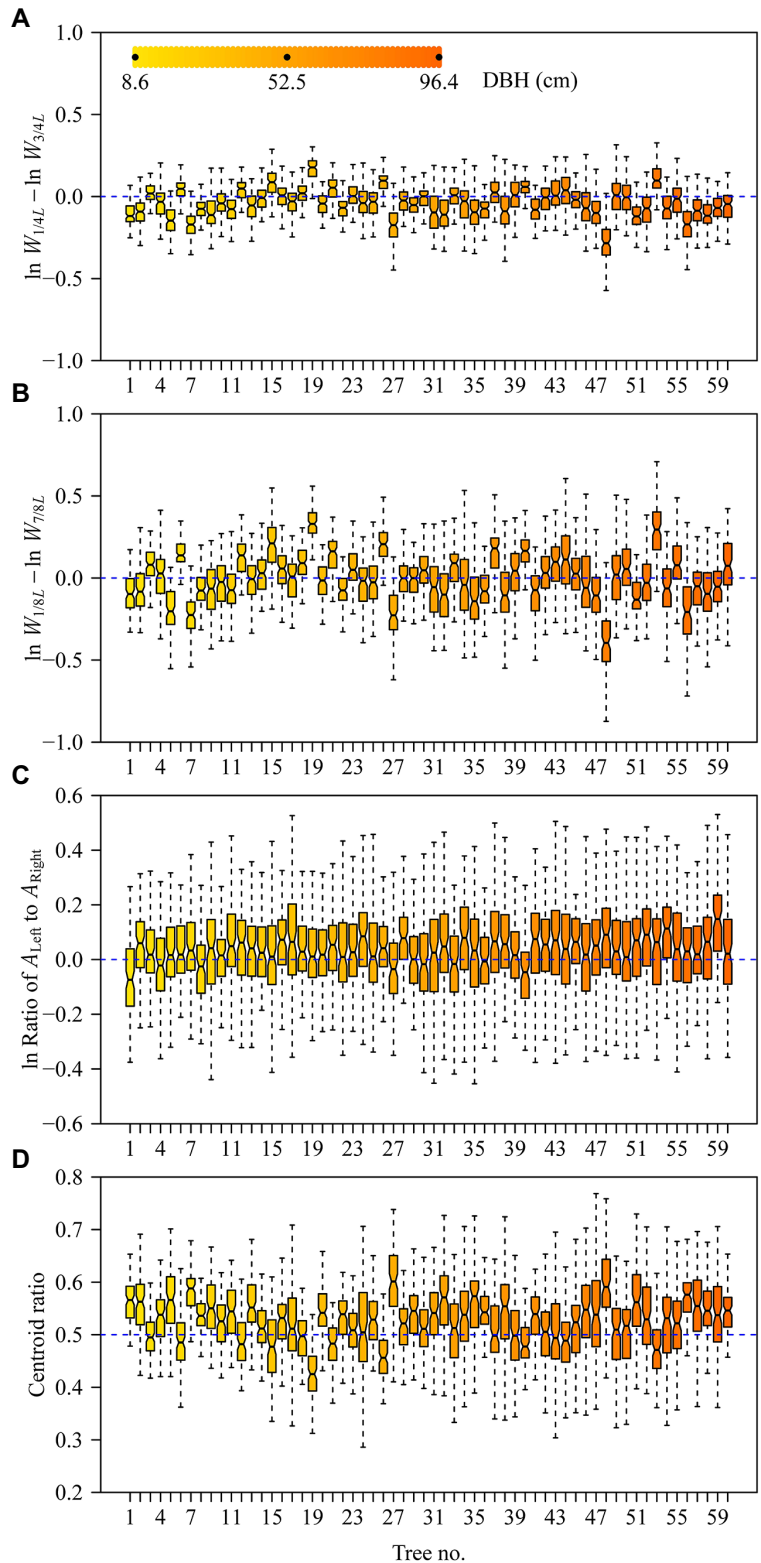
Boxplots of the logarithm of leaf proximal ratio index **(A)**, logarithm of leaf distal ratio index **(B)**, logarithm of the area ratio of the left side to the right side **(C)**, and centroid ratio **(D)**. The notches of boxes represent the medians, and the colors of boxes reflect their DBH values (see panel **A**).

The DBHs of the trees examined over the course of this study ranged from 8.6 to 96.4 cm. Tree height ranged from 3 to 32 m. Table 2 shows the influence of DBH and site on leaf size, shape, and LMA. Leaf size and the ratio of leaf width to length (RWL) tend to increase with increasing DBH (Figures 2A,C), whereas LMA decreases with increasing DBH (Figure 2B). DBH did not significantly affect the leaf ellipticalness index (EI; Figure 2D; Table 2), which appears to result from a random effect. There were large variations among the sites for EI, leaf area, LMA, and RWL, with ρ ranging from 0.1832 to 0.5056 (Table 2). For other leaf shape indices, DBH had a statistically significant effect on the proximal ratio index (PRI), and the random site effect was very minor (Figures 3A–C; Table 2). With increasing DBH, the leaf centroid did not shift closer to the leaf apex or to the leaf base (Figure 3D; Table 2), that is, leaves morphologically maintain an oboval shape rather than an elliptical shape regardless of tree size. The DBH did not significantly affect the distal ratio index (DRI) or the area ratio of the two sides of leaves (AR), and the random site effect was very minor, with ρ < 0.1. The intercept of ln AR was not significant (*p* > 0.05), which indicated that there was no significant difference in area between the two sides of leaves (Table 2).

**Table 2:** Fitted results of the linear mixed model to eight leaf size and shape indices.

Item	Estimate		Significance (*P*)		Standard deviation		Intraclass correlation coefficient
	Intercept	DBH	Intercept	DBH	Site	Residual	
Leaf area (m^−2^)	6.780850	0.107641	<0.05	<0.05	3.5449	5.6104	0.2853
LMA (g m^−2^)	189.151290	−0.151780	<0.05	<0.05	14.9116	31.4880	0.1832
Leaf width/length	0.676246	0.000298	<0.05	<0.05	0.0437	0.0871	0.2009
Leaf ellipticalness index	0.984667	−0.000079	<0.05	>0.05	0.0382	0.0377	0.5065
ln *W*_1/4*L*_/*W*_3/4*L*_	−0.031559	−0.000278	<0.05	<0.05	0.0134	0.1179	0.0128
ln *W*_1/8*L*_/*W*_7/8*L*_	−0.011575	0.000253	>0.05	>0.05	0.0305	0.1977	0.0233
ln *A*_Left_/*A*_Right_	0.027439	0.000183	>0.05	>0.05	0.0224	0.1611	0.0190
Centroid ratio	0.523712	0.000041	<0.05	>0.05	0.0053	0.0692	0.0059

**Figure 4:**
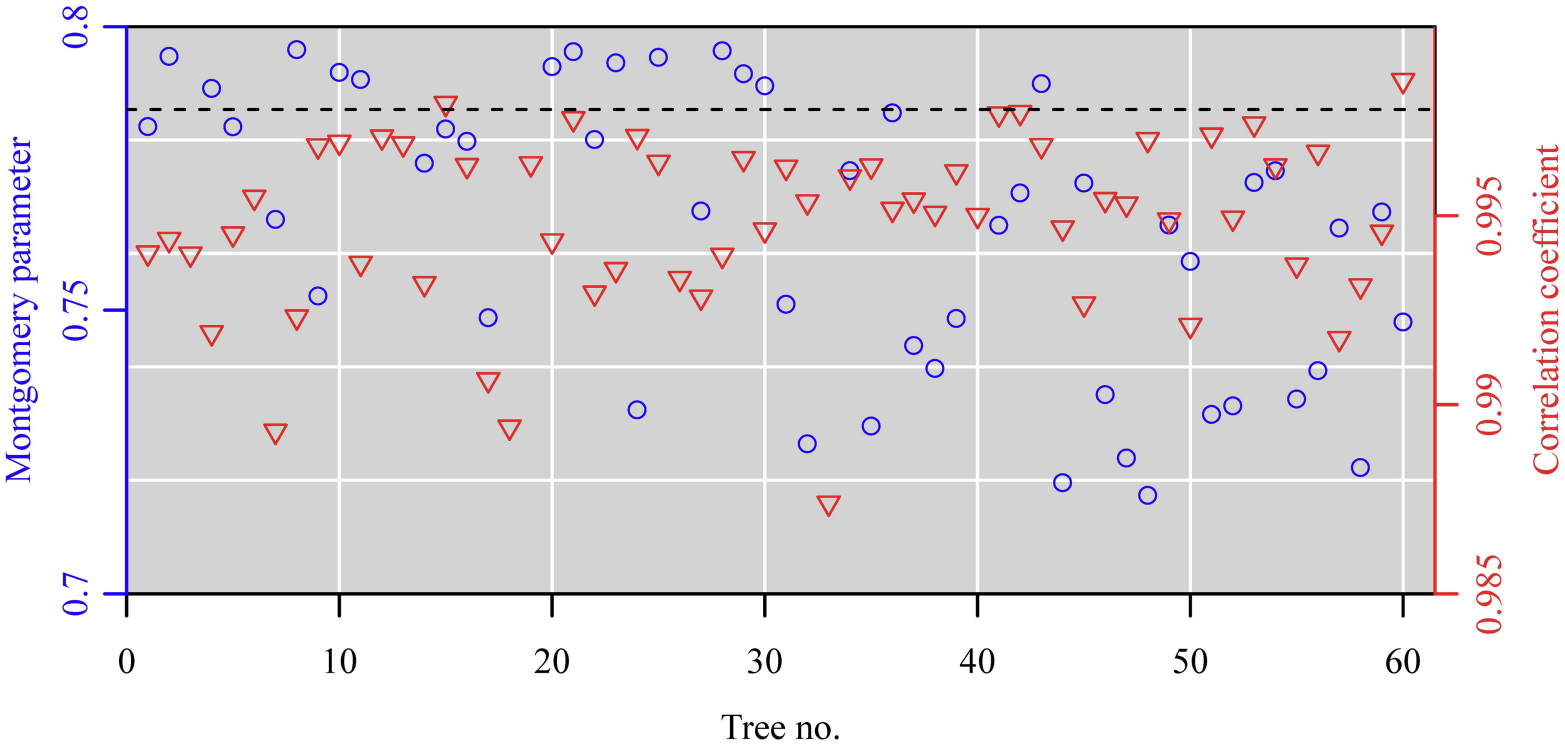
The estimates of the Montgomery parameters (blue open circles) and correlation coefficients (red open lower triangles) for the leaves sampled from each of the 60 trees. Each Montgomery parameter (MP) was estimated as a proportionality coefficient for leaf area = MP × leaf length × length width on a log–log scale, and the correction coefficient was used to reflect the linear degree between leaf area and the product of leaf length and width on a log–log scale.

**Figure 5:**
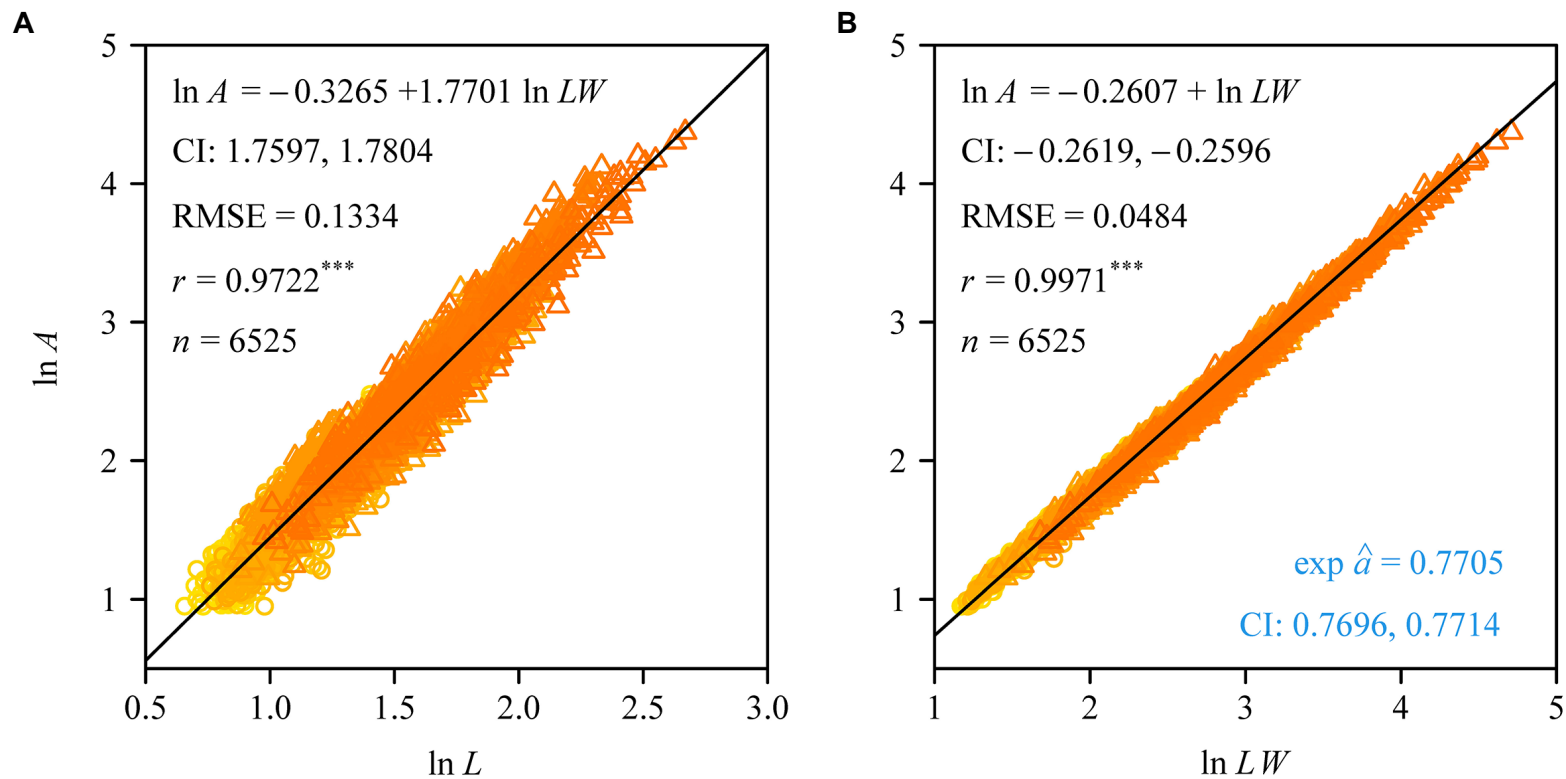
Fitted results to the data of leaf area vs. leaf length **(A)**, and the data of leaf area vs. the product of leaf length and width **(B)**. In panel **(A)**, CI represents the 95% confidence intervals of the slope; in panel **(B)**, CI represents the 95% confidence intervals of the exponential of the intercept, i.e., the Montgomery parameter’s CI. RMSE is the root-mean-square error of the linear regression; *r* is the correlation coefficient, with three asterisks indicating *p* < 0.001; *n* is the sample size, i.e., the number of the pooled data.

**Figure 6:**
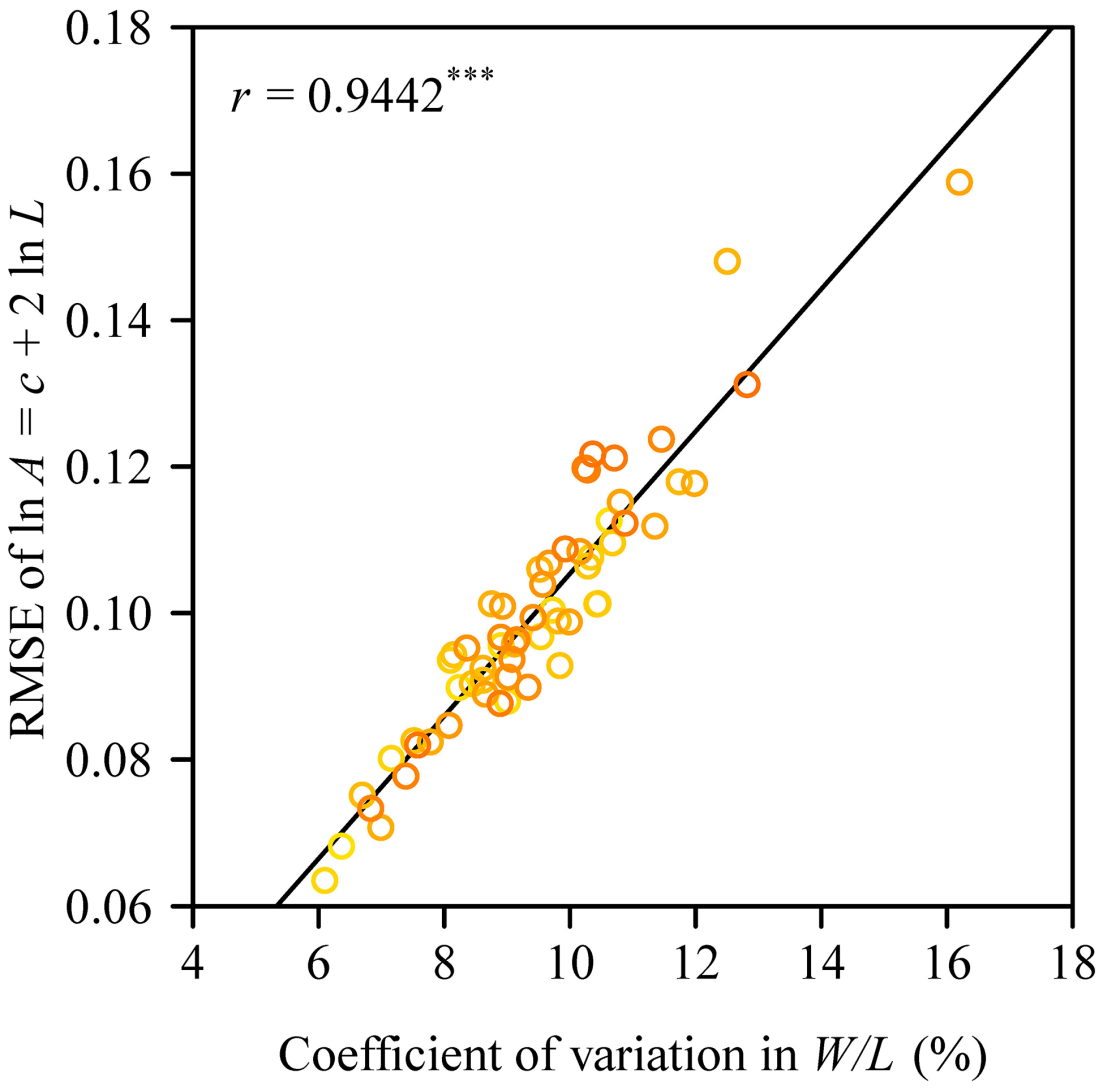
Correlation between the goodness of fit based on the principle of similarity (assuming a square relationship between leaf area and leaf length) and the coefficient of variation in the ratio of leaf width to length. The *y*-label represents the root-mean-square error of the linear regression for the relationship between leaf area and the square of leaf length on a log–log scale; and *r* is the correlation coefficient, with three asterisks indicating *p* < 0.001.

The Montgomery equation (ME) was found to be valid for the leaves of each tree with a correlation coefficient *r* ranging from 0.985 to 0.999. The estimated Montgomery parameter (MP) ranged from 0.7 to 0.8, and exceeded π/4 for 13 out of the 60 trees examined (Figure 4). Using the pooled data of the 60 trees, there was a significant log–log linear relationship between leaf area and leaf length on a log–log scale, and that between leaf area and the product of leaf length and width (Figure 5). However, the latter had a higher goodness of fit for a <0.05 RMSE than the former with a >0.13 RMSE. The 95% confidence intervals of the slope did not include 2 (Figure 5A), which indicated that the principle of similarity did not hold true for this oak species. There was a strong correlation between the goodness of the fit of the *A* vs. *L*^2^ data on a log–log scale and the coefficient of variation of RWL (Figure 6). A smaller RMSE corresponded to a better goodness of fit and a lower coefficient of variation in RWL. Therefore, overall, the ME was found to be valid for calculating leaf area both at the individual tree level and for the pooled data across all individuals that were examined. Although the estimated MP values differ across individual trees, these values varied over a small range, which resulted in a <0.05 RMSE by fitting the pooled leaf data of the 60 trees.

## Discussion

### Leaf Size and LMA of *Quercus pannosa*

The data presented here reveals a trend in which leaf size increases with increasing tree size (as measured by DBH), which is not in accord with previous reports of the opposite trend (England and Attiwill, 2006). A number of possible explanations for this contradiction become apparent. However, we believe that it might result from the differences in the conduit sizes (vessel diameters) with increasing overall tree size reflecting an adaptation to extreme alpine environments. The number of days of frosts per year is more than 150 days in the study area. *Quercus pannosa* has evolved a special diffuse porous anatomy with more comparatively small diameter conduits capable of avoiding fatal winter embolism across multi-year vessel cohorts (Yang et al., 2020), since freezing can cause xylem cavitation for alpine trees (Mayr et al., 2007). However, narrower vessels also have a greater resistance to water transport, which limits the rate at which water can be delivered to leaves high in the canopy. The mean vessel diameter of newly formed vessels in larger *Q. pannosa* trees tends to be larger than that of newly formed vessels in smaller trees. It is possible therefore that this ontogenetic anatomical shift in vessel size permits the development of larger leaves (see Figure 2A). Future research in this area is required.

Turning attention to the morphometrics of leaves, it is important to note that LMA reflects the leaf-level cost of light interception (Poorter et al., 2009), and serves as an important indicator of plant ecological strategies (Westoby et al., 2002). A high LMA and long leaf lifespan dimension signifies slow turnover of plant components, long nutrient residence times, and slow response to favorable growth conditions (Westoby et al., 2002). In light of the extreme growth environment of *Q. pannosa*, we speculate that the leaves of this species are typically in a state of water deficiency as a consequence of the comparative narrow vessels in their wood. A drought environment usually correlates with large LMA values (Poorter et al., 2009), and the mean LMA of *Q. pannosa* ranges between 100 and 250 g m^−2^ (Figure 2B), which is larger than that previously reported for other evergreen trees, that is, 50–50 g m^−2^ as is reported by Poorter et al. (2009). Our results are inconsistent with those of England and Attiwill (2006) who found that SLA (the reciprocal of LMA) of a *Eucalyptus* species decreases with increasing tree age, which translates in a trend of increasing LMA. The present work shows that LMA decreases with increasing DBH. This can be explained based on the differences in the conduit sizes (vessel diameters) with increasing overall tree size reflecting an adaptation to an extreme alpine environment. Because larger trees have larger mean vessel diameters in their newly formed vessels than smaller trees, water transport in larger trees is relatively better than in younger trees, i.e., the leaves of small trees are in a state of water deficiency, and thus have larger LMA values.

### Leaf Shape of *Quercus pannosa*

**Figure 7:**
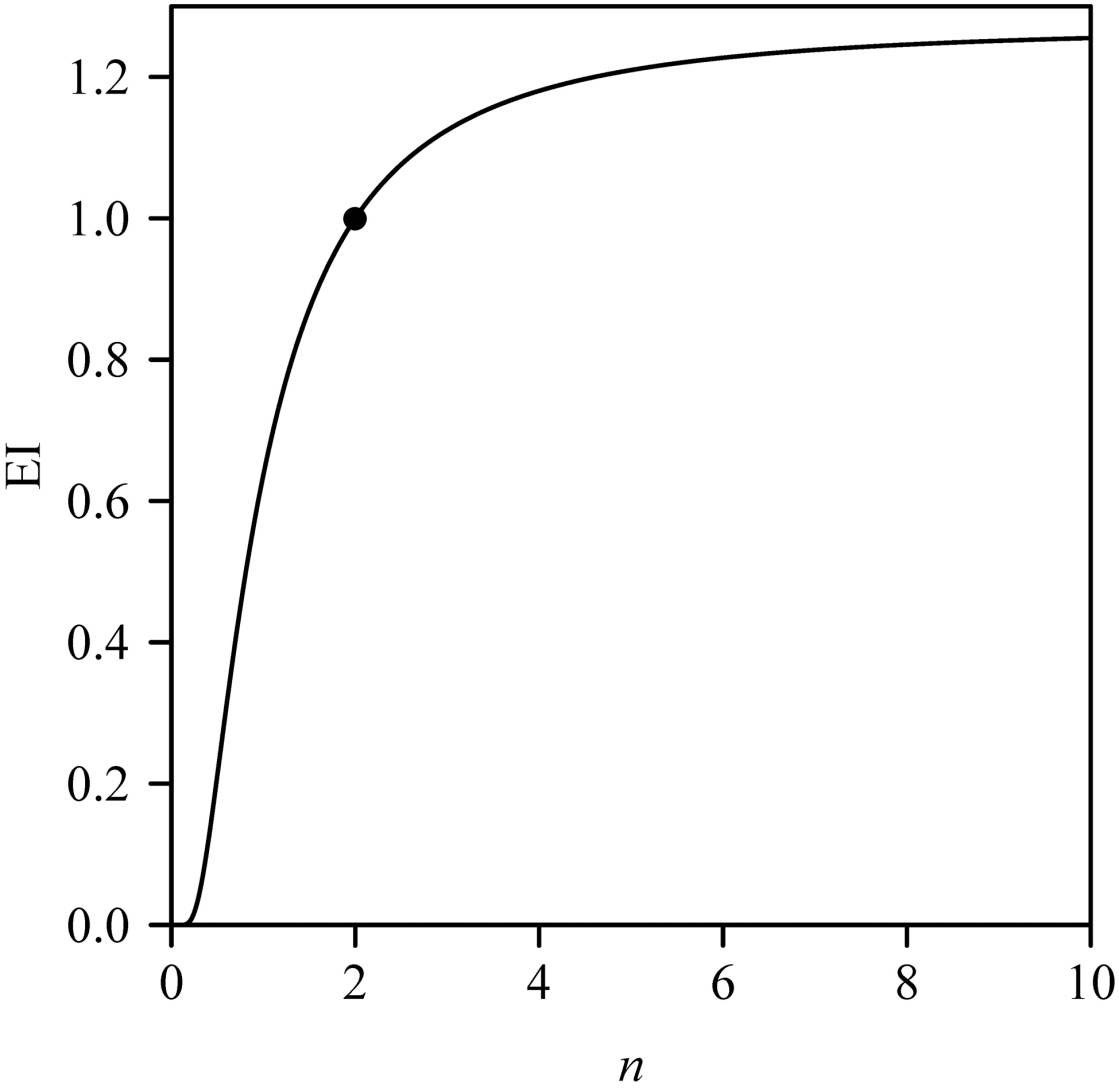
Leaf ellipticalness index (EI) varying with the *n* values in the superellipse. The point corresponds to the EI when *n* = 2.

Previous studies have shown that the Montgomery parameter (MP) of most leaves ranges from 1/2 to π/4 (Shi et al., 2019; Yu et al., 2020; Schrader et al., 2021). Even in the case of the oblong and oblate leaf-shapes examined by Schrader et al. (2021), MP tends to be <π/4. However, in the present study, 13 out of a total of 60 MP values were larger than π/4 (Figure 4). In addition, most of the mean centroid ratios were numerically >0.5 (Figure 3D). These features indicate that the leaf shape of *Q. pannosa* is not a standard ellipse. Indeed, visual inspection (Figure 2D) shows that the leaves of this species are not ellipses, a feature that is numerically quantifiable by virtue of the leaf ellipticalness index (EI), which is either >1 or <1. Thus, the leaf-shape of *Q. pannosa* may be a superellipse rather than an ellipse (Gielis, 2003; Li et al., 2021a), as defined by the formula


$${\left|x/\alpha \right|}^{n}+{\left|y/\beta \right|}^{n}=1,$$

where *x* and *y* are the planar coordinates of a superellipse, and *n* is a parameter determining the shape of the superellipse. The area formula of a superellipse (Huang et al., 2020) is


$$A=\frac{4^{-1/n}\sqrt{\pi }\;\Gamma \left(1+1/n\right)}{\Gamma \left(0.5+1/n\right)}LW,$$

where Γ is the gamma function. With *n* −>∞, the superellipse will approximate a rectangle, so MP −> 1 and EI −> MP/(π/4) ≈ 1.27. Figure 7 shows that EI is a sigmoid function of *n*, and has an asymptotic value. This suggests that *Q. pannosa* might produce approximately superelliptical leaves. In this regard, Li et al. (2021a) have demonstrated the existence of superelliptical leaves in nature for two Magnoliaceae species.

Finally, it is noteworthy that the ratio of leaf width to length (RWL) increases with tree size (Figure 2C; Table 2), which differs from the leaves produced by other tree species (England and Attiwill, 2006). This phenomenology might be related to water deficiency. The relationship between conduit size and tree size with the corresponding influences on leaf size and shape deserves further investigation.

## Conclusion

Bigger trees (as measured by DBH) of *Q. pannosa* tend to have larger and broader leaves. The LMAs for the 60 trees are much larger than those reported for other evergreen tree species. We conclude that this phenomenology reflects hydraulic limitations resulting from adaptions to the cold alpine environment in which this species of oak grows. The mean leaf centroid position exceeds the midpoint of leaf length, but the centroid does not shift closer to the leaf apex with increasing tree size. There is no significant difference in the lamina area of the two sides of leaves, which indicates a bilateral symmetry for *Q. pannosa* leaves. The relationship between leaf area and length does not support the principle of similarity, which postulates that the area of an object is proportional to the square of its length. Our results show that the principle of similarity depends on the extent of variation in the ratio of leaf width to length (RWL). A larger coefficient of variation in RWL obtains a larger prediction error when the principle of similarity is used to calculate leaf area. In contrast, the variation in leaf shape does not affect the validity of the Montgomery equation in calculating leaf area based on leaf length and width. The effect of tree size on leaf area can be neglected when using the Montgomery equation could be better related to the altitudes at which *Q. pannosa* grows and its hydraulic limitations resulting from adaptions to the cold alpine environment.

## Data Availability Statement

The original contributions presented in the study are included in the article/Supplementary Material, further inquiries can be directed to the corresponding author.

## Author Contributions

JM, KN, and PS designed this work, analyzed the data, and wrote the manuscript. JM, LL, ZF, and YL carried out the experiment. All authors contributed to the article and approved the submitted version.

## Funding

JM was supported by the Economic Research Center of State Forestry and Grassland Administration of China (No. JYC2020-YN01) and National Natural Science Foundation of China (No. 32171539).

## Conflict of Interest

The authors declare that the research was conducted in the absence of any commercial or financial relationships that could be construed as a potential conflict of interest.

## Publisher’s Note

All claims expressed in this article are solely those of the authors and do not necessarily represent those of their affiliated organizations, or those of the publisher, the editors and the reviewers. Any product that may be evaluated in this article, or claim that may be made by its manufacturer, is not guaranteed or endorsed by the publisher.

## Footnotes

^1^ http://data.cma.cn/
